# Evaluation of the Effect Coronavirus Lockdown had on Chronic Disease Management Care in Pediatrics: A Survey of Jordanian Pediatricians

**DOI:** 10.1155/2022/8710176

**Published:** 2022-10-06

**Authors:** Salma M. Burayzat, Muna Kilani, Redab Al-Ghawanmeh, Mohnad Odeh, Omar Assi, Karem H. Alzoubi

**Affiliations:** ^1^Department of Pediatrics, Faculty of Medicine, The Hashemite University, P.O. Box 330127, Zarqa, 13133, Jordan; ^2^Department of Clinical Pharmacy and Pharmacy Practice, Faculty of Pharmaceutical Sciences, The Hashemite University, P.O. Box 330127, Zarqa, 13133, Jordan; ^3^Department of Pharmacy Practice and Pharmacotherapeutics, College of Pharmacy, University of Sharjah, Sharjah, UAE; ^4^Department of Clinical Pharmacy, Jordan University of Science and Technology, Irbid, Jordan

## Abstract

The outbreak of the COVID-19 pandemic puts a great strain on the healthcare system, as the national and global infection rates increased rapidly. Efforts were devoted to minimizing the effects of the spreading pandemic without overwhelming the already stretched healthcare system. The study objective was to establish how coronavirus lockdown was affecting chronic disease care among pediatric patients admitted to hospitals in developing countries. For that purpose, a cross-sectional survey of registered pediatricians was carried out. Findings showed that the mortality rate from COVID-19 in children seemed to be low. However, children with chronic illnesses were likely to be gravely affected by the disturbance of repetitive healthcare services. About 79% of pediatricians treated a child with a chronic condition during the lockdown. Furthermore, 15% of patients with chronic diseases visiting pediatricians exhibited uncontrolled emerging complications. In addition, 9% of pediatricians reported one to five fatalities among children under their care due to delay or lack of appropriate medical care in the period of the lockdown. Residents (48.9%) reported a significantly (*p* < 0.001) higher proportion of providing face-to-face services compared with consultants (17.9%) and specialists (13.6%). In conclusion, the care of chronically ill children should be taken into consideration when implementing lockdown and/or social distancing, thus minimizing the negative effects of lockdown and/or social distancing on children with chronic diseases.

## 1. Introduction

The initial discovery of coronavirus, also known as (SARS-COV-2), began from patients suffering from pneumonia. The first case of coronavirus was reported in December 2019 in Hubei province, China. The World Health Organization (WHO) pronounced the spread of this virus on 30^th^ January 2020 and later in March 2020, and the disease was declared as a global pandemic [1]. By April 2022, over six million individuals died with COVID-19. Lockdown and social distancing were the major practices implemented in an effort to contain the pandemic. This has led to drastic changes in daily routines, eating habits, and physical activities [2, 3].

Caregivers of children suffering from chronic diseases are challenged with their daily routines of exercise, diet, and the need for regular follow-up. Strict lockdown and social distancing related to COVID-19 have rendered coping with existing chronic diseases much more challenging. This is related to the lack of access to health facilities and healthcare providers, especially in developing countries [4]. The influence of COVID-19 on the healthcare system was tremendous and multifaced. There was an overwhelming demand for healthcare system services, workers, and health facilities. During the different phases of complete and partial lockdown, healthcare access was compromised. While some of the policies adopted by the governments were strict, they affected the unique patient population. This is especially true for children with chronic diseases who found it difficult to reach their caring specialists or health care providers [5]. On the other hand, indirect effects of epidemics such as in 2003, the Severe Acute Respiratory Syndrome (SARS) epidemic [6], and the Ebola virus epidemic of 2014 in West Africa were seen through history [7, 8]. Meanwhile the pediatric patients with chronic diseases have shown some deterioration in management and follow up during lockdown [9].

The long-lasting impact of the disruption of care of patients with chronic diseases is likely to surpass the duration of the COVID-19 pandemic. World Health Organization (WHO) reported that irrespective of the disease prognosis, patients presenting with different co-morbidities have a high risk for severe COVID-19 disease course and complications [10]. In pediatric patients with chronic diseases such as chronic lung disease, primary immune deficiency, chronic renal disease, and neurological disorders, the risk for morbidity and mortality of COVID-19 disease is increased [11]. In order to provide the optimum management for those children, the necessary precautions should be planned for, and follow-up pathways should be specified [11].

The current study was aimed to assess the effects of lockdown on maintaining an acceptable chronic disease follow up in pediatric population. This subgroup of patients might face a more difficult course in the management and follow up especially taking into consideration that telemedicine is less reliable in this population [9]. The studies in this subgroup are scarce and more focused on adult population rather than pediatrics. [12]. This study should establish how coronavirus lockdown was impacting chronic disease management in pediatric patients admitted to hospitals in a developing country setting.

## 2. Methods

### 2.1. Research Design

A cross-sectional study based on online survey.

### 2.2. Sampling

All Jordanian pediatricians (*n* = 840), who are registered in the Medical Union and the Jordanian Pediatric Society, were contacted through their registered official e-mail and phone number. Registration in the union is an obligation to practice pediatrics in Jordan. The sample included mainly general pediatricians from all medical sectors; governmental entities, universities, and the private sectors. Pediatricians with subspeciality are scares in Jordan leading to the fact that most children with chronic diseases are followed up by general pediatricians. Moreover, pediatricians in Jordan are subdivided into residents, who are under training (yet in the COVID pandemic compromised the majority of working physicians), specialists, and consultants. Pediatric specialists and consultants carry out the same duties but with difference in years of experience where consultants should have more than 10 years of experience in their field. The sample size of this study was calculated through the sample size calculator. A representative sample size of pediatricians in Jordan was calculated to be 264 participants providing 95% confidence interval and 5% margin error for a population of 840 active pediatricians.

### 2.3. Instruments and Data Collection

The study instrument was pilot tested on a sample of 10 pediatricians. Statisticians approved the questionnaire after the pilot testing. The ten pilot test participants were included in the study sample as there were no changes done to the questionnaire. The study was an open-ended questionnaire study comprised of valid cross-examinations to capture the responses from selected research contributors. The questionnaire was constructed using Google Forms. An online link to the survey was sent to registered pediatricians through their official emails and phone numbers. The questionnaire was distributed on June 16, 2020, and the form was closed to responses when we reached the desired sample size on September 30, 2020. The response rate was 31.4%. Data were retrieved in Excel and analyzed using SPSS version 25 (SPSS Inc. Chicago, Illinois, USA).

### 2.4. Ethical Considerations

This research activity was revised then attained an endorsement from the IRB committee, Faculty of Medicine at Hashemite University, Jordan. The objectives of the study were explained at the beginning of the questionnaire to all participating pediatricians.

### 2.5. Data Analysis

Data captured during this study were inspected using both quantitative and qualitative methods. Quantification of the research was carried out through expressing responders' answers by frequencies and percentages. Univariate analysis was carried out to test the association between the level of practice of responders, and characteristics of pediatric health services provided to patients during the pandemic. In cases where the chi-square test for association showed statistically significant relationships (*p* < 0.05), Cramer's *V* coefficient was used to assess the magnitude and strength of nominal-by-nominal relationship. The following “crude estimate” values were considered for interpreting the strengths of relationships. The absolute value of the coefficient of <0.19 was considered a negligible relationship, 0.2–0.29 was considered a weak relationship, and 0.30–0.39 was considered a moderate relationship. In cases where a statistically significant association and moderate strength relationships were demonstrated, further post hoc analysis was carried out through a cell-by-cell comparison approach. The adjusted standardized residual was used to provide post hoc evidence of association. If an adjusted standardized residual is positive, it indicates that there are more observed frequencies than expected frequencies. If an adjusted standardized residual is negative, it indicates that there are less observed frequencies than expected frequencies given the null hypothesis of independence. If the standardized residual had an absolute value > 3, then the lack of independence is further approved. This indicates that the variables tested are either positively or negatively associated as confirmed by the post hoc evidence of association.

## 3. Results

A total of two hundred sixty-four participants filled the online survey, providing 95% CI and 5% margin error for 840 active Jordanian pediatricians.  Table 1 illustrates a summary of the basic demographic characteristics of the participants. Participants were almost equally distributed across different levels of practice. Consultants were 95 (36%), specialists were 81 (30.7%), and residents were 88 (33.3%). The most frequent age category from the study was 31–40 years (*n* = 120, 45.5%). The number of male participants was 148 (56.1%). Respondents from Ministry of Health hospitals *n* = 126 (44.7%) returned to most of the polls. Residents were the largest group of responders from the Ministry of Health (79.5%). Respondents from the private sector constituted 28.4% (*n* = 75), while from the Royal Medical Services 14.8% (*n* = 39) responded to the survey. Only *n* = 24 (9.1%) of the responses came from university hospitals. Most of the responders practiced pediatrics in the capital city-Amman (71.2%, Table 1 and Supplementary Table 1).

Specific effects of COVID-19 lockdown procedures in relation to level of practice are summarized in Table 2. Due to official COVID-19 related procedures, 26.9% (*n* = 71) of the respondents had their facility of practice exclusively dedicated for treating COVID-19 patients. Meanwhile, most pediatricians *n* = 217 (82.2%) continued practicing during the lockdown period, and 20.5% (*n* = 54) of the pediatricians had COVID-19 positive patients to attend to. About, 55.6% of all patients who were diagnosed with COVID-19 came from the Ministry of Health hospitals. The Ministry of Health had the highest burden of patients during the lockdown. For example, the Ministry of Health's proportion was 75% among all other sectors for managing more than 30 patients per pediatrician per day. Residents (48.9%) reported a significantly (*p* < 0.001) higher proportion providing face-to-face services compared with consultants (17.9%) and specialists (13.6%). In comparison, 34.7% of consultants provided remote services compared with 16.1% of specialists, and only 1.1% of the residents (*p* < 0.001, Table 2).

Regarding the care of chronically ill children, 79% of pediatricians treated at least one child with a chronic condition, and all of them had at least one patient with a status change during the lockdown period. Almost half of such patients were stable yet needed closer follow-up and approximately 15% of patients with chronic diseases visiting pediatricians were uncontrolled and had emerging complications. The emerging complications were related to the chronic diseases the patients suffered from either flare-ups or complications due to not adhering to management. Of respondent pediatricians, 30% felt comfortable contacting the primary care physician. Among those, a good connection with the primary care team was observed, as 41.3% of respondents contacted the primary care team to manage patient care. Moreover, 43.2% of respondents reported that it was easy to contact the primary care physician. Unfortunately, according to pediatricians, 84% of caregivers faced difficulties in getting a patient prescription. Only 4.9% of caregivers were never reluctant to provide medical care during the lockdown. Even in emergencies, only 8.7% of caregivers had never avoided going to the emergency department during the lockdown (Table 2). About 31% of pediatricians participating in this survey reported a loss of a chronically ill patient, not due to COVID-19 diagnosis, and approximately 9% of these pediatricians have lost more than five patients. After the experience with COVID-19 in Jordan, 40% of active pediatricians disagreed with choosing tertiary hospitals as quarantine hospitals for COVID-19 patients, and 63.3% recommended trying to pick other establishments outside large cities with less negative impacts on patients. In comparison, 21.2% agreed with the same procedures.

The effect of levels of experience was studied in this questionnaire. Statistically significant differences in responses to the questionnaire were noticed in three questions. However, the magnitude and strength of association between answers and the level of specialty were weak. Female specialists were significantly more than males. Male residents were significantly more than females. However, the strength of such association was negligible (*χ*^2^ (4) = 18.06, Cramer's *V* = 0.185, *p* = 0.001, Supplementary Table 2). A significantly higher proportion of all levels of specialty practiced pediatrics during the lockdown period. However, the association between variables was negligible, Cramer's *V* = 0.171. The association with practice level was significant *χ*^2^ (4) = 15.62, *p* = 0.016. On the other hand, a higher proportion of respondents confirmed that patients were stable yet needed closer follow up; however, the association was negligible, Cramer's *V* = 0.172, Supplementary Table 2.

A moderate strengh statistically signficant association between the place of practice and the level of practice was noticed (*p* < 0.001). Residents (79.5%) at the Ministry of Health hospitals were more than the expected frequencies (Standardized Residual = 4.3). The observed frequencies at the private sector were less than expected (Standardized Residual = −3). The highest proportion of consultants was noticed at the private sector (38.9% of consultants). The lowest proportion of specialists was noticed at university hospitals (6.2%), Supplementary Table 3.

Among all levels of practice, residents reported significantly (*p* < 0.001) higher proportion (28.4%) of seeing more than 30 patients per day compared with specialists (6.2%) and consultants (6.3%). The observed frequencies were more than the expected frequencies (Standardized Residual = 3.8, Supplementary Table 4). Delivering services by consultants through remote means, such as phone or Facebook, showed the highest level of association compared with resident and specialists (*p* < 0.001, Cramer's *V* = 0.33 and the Standardized Residual = 3.9, Supplementary Table 5).

Despite relatively weak association (Cramer's *V* = 0.236), residents reported the highest frequency (19.3%) for fatalities, where consultants and specialists reported 3.2% and 3.7%, respectively. The observed frequencies were more than the expected frequencies (Standardized Residual = 3.4, Supplementary Table 6).

## 4. Discussion

In Jordan, as in many other developing countries, healthcare is provided by the public and private sectors, yet the public sector carries the heaviest burden; thus, most of the respondent pediatricians were from the public sector as well as 30% of COVID-positive patients at the time of the study. In Jordan, a total of 106 public and private hospitals are equipped with more than 12,000 beds. About 67% of these beds are provided by the public sector [13]. When COVID-19 posed a considerable burden on many countries, governments worldwide had to face the challenge and flatten the infection curve. These efforts were aimed towards minimizing the direct effect of the rapidly spreading pandemic without overwhelming the already stretched health care system. Governments should not overlook other health services, mainly as pandemics are the threat of today's world. Notably, in the countries with strict policies and lockdowns, there were reports of disrupted primary healthcare facilities such as labor and delivery services, immunization, HIV and TB care, dialysis, and cancer treatment [14].

One of the most troublesome indirect effects of lockdown was the restriction on routine physician consultations. Consequently, children suffering from chronic illnesses, even if stable, could not access the usual values of care due to the restrictions of movement and deployment of healthcare personnel to the frontline of the COVID-19 contamination. These strict governmental policies impacted significantly the patients suffering from chronic diseases that require revisits as well as prescription refills. These impacts denied access to healthcare facilities and attending doctors [6, 15]. As a sequence of these policies, pediatricians who had to follow these chronically ill patients apart from their treating physicians reported that around 80% of these chronically ill children had a change of status. Although 40% of the pediatricians in this study were able to contact the subspecialists responsible for the chronically ill children in their care, 30% reported a loss of at least one of these children. Moreover, around 9% reported the loss of more than five children with chronic illnesses not due to COVID-19 infection but due to the improper management of these children in relation to the lack of the specialized facilities as these facilities were locked down for quarantine for COVID-19 patients.

Multiple studies documented that complicated COVID-19 cases had pre-existing medical conditions such as chronic lung disease, cardiovascular disease, immunosuppression, obesity, and neurologic conditions. The risk of COVID-19 infection and the severity of illness in children with pre-existing conditions can be greatly reduced via abiding and adhering to proper physical distancing, hand hygiene, mask use, attending to underlying medical conditions, and management of surfacing complication under doctors' guidance [16–18].

During the lockdown drastic measures, one of the solutions for establishing patient-physician contact was telemedicine. Among participants of this study, only 18% of physicians reported using social media or telemedicine to contact their patients. Virtual visits have been approved to decrease personal medical visit up to about 50% across specialties. Aydemir and colleagues reported in their study that despite poor telemedicine experience, there was an overall patient and physician satisfaction with using telemedicine in follow-up visits of chronically ill children [19].

Virtual consultations reduce deaths besides lessening the number of days a patient stays in the hospital even if the patient is in life-threatening care situations. In developed countries, the presence of a practical infrastructure led to well-established app-based medical services [20–22]. On the other hand, Mubaraki and colleagues addressed the issue of telemedicine disadvantages in their research and reported that it was difficult to reach a definitive diagnosis through remote consultations. They also reported that the fear of sudden practice changes caused by the COVID-19 was an issue that led the physicians to be prudent while using telemedicine in their practice during the pandemic [16, 23].

The change in the health status of these children could be attributed to one of the following factors during the lockdown; (1) patients and caregivers face difficulties reaching emergency rooms, (2) they fear that the children might get infected, or (3) they are unable to get their prescription medications, and most of the time a combination of all. Myonihan and colleagues highlighted the long-term impacts of missed care of those without COVID-19 disease during the pandemic. It was recommended that public campaigns should urge public and caregivers to seek medical care when needed as evidence by excess population mortality that was demonstrated in several studies [24].

The direct and indirect effects of lockdown and quarantine policies should always be thoroughly studied before imposing strict policies and defense orders. Sixty-three percent of the participant pediatricians disagreed with choosing large, specialized tertiary hospitals for quarantine of patients simply because they have a positive COVID-19 test result. This is because choosing large, specialized tertiary hospitals for quarantine has a significant negative impact on the continuity of care for the chronically ill children who were being followed in these facilities.

COVID-19 is likely to leave behind a long-term legacy and sequel that can only be revealed in the future. However, learning about failing policies along the pathway might encourage better healthcare-oriented decisions to minimize the impact of this aggressive pandemic and control its direct and indirect damages. Governments should always consider the indirect and remote consequences of their health policies when imposing strict measures during extraordinary situations, as the indirect impact of these policies might prove to be more devastating than the pandemic itself. To minimize these indirect sequelae of lockdown, state agencies in developing nations should recognize and legitimize remote healthcare distribution in addition to virtual consultation.

The limitations of this study can be attributed as the cross-sectional design of the survey, which was directed at Jordanian pediatricians and the fact that there were no previous studies on the health care services status in Jordanian Hospitals for purposes of comparison.

## 5. Conclusion

The health care policies and decisions taken to overcome the sequels of pandemics must always take into consideration preserving health care services for patients with chronic diseases who depend on these health care facilities for a better quality of life.

## Figures and Tables

**Table 1:** Demographics and characteristics of responders.

Characteristic		Frequency	Total no. (%)	*p* value
Level of practice.	Consultant	95	**36.0**	
	Resident	88	**33.3**	
	Specialist	81	**30.7**	

Age	<30	53	**20.1**	
	31–40	120	**45.5**	
	41–50	43	**16.3**	
	>50	48	**18.2**	

Gender	Female	114	**43.2**	**0.001** ^§^
	Male	148	**56.1**	
	Prefer not to answer	2	**0.8**	

Place of practice	Ministry of health	126	**47.7**	**0.001** ^£^
	Private sector	75	**28.4**	
	Royal medical services	39	**14.8**	
	University hospital	24	**9.1**	

City of practice	Amman	188	**71.2**	
	Irbid	23	**8.7**	
	Balqa	19	**7.2**	
	Zarqa	14	**5.3**	
	Karak	8	**3.0**	
	Others	12	**4.3**	
			

^§^Significant difference according to level of practice, with week magnitude; Supplementary Table 2. ^£^Significant difference according to level of practice, with moderate magnitude and standardized residual absolute value > 3; Supplementary Table 3.

**Table 2:** Effect of COVID-19 pandemic on health services in Jordan.

Variable	Choices	N	(%)	*p* value
Practice facility dedicated for COVID-19	No	193	73.1	**0.058** ^*∗*^
	Yes	71	26.9	

Attend to COVID-19 positive patients	No	210	79.5	**0.178** ^*∗*^
	Yes	54	20.5	

Practiced pediatrics during the lockdown	No	47	17.8	**0.021** ^§^
	Yes	217	82.2	

The number of patients seen in different changed during lockdown	Largely decreased	100	37.9	**0.240** ^*∗*^
	Decreased	79	29.9	
	Did not change	66	25.0	
	Increased	14	5.3	
	Largely increased	5	1.9	

The number of patients seen/day during lockdown	0	32	12.1	**0.001** ^£^
	1–10	109	41.3	
	11–20	59	22.3	
	21–30	28	10.6	
	>30	36	13.6	

Method of practice during lockdown	Both ways	146	55.3	**0.001** ^£^
	In-person; face to face	71	26.9	
	Remotely, by phone, Facebook etc	47	17.8	

Have seen children who have been already diagnosed with chronic illnesses	No (%)	55	20.8	**0.203** ^*∗*^
	Yes (%)	209	79.2	

The health condition of most of the chronically ill children seen during lockdown	Controlled on given management	65	24.6	**0.016** ^§^
	Not applicable	28	10.6	
	Stable, yet need closer to follow up	132	50.0	
	Uncontrolled with emerging complications	39	14.8	

Feeling comfortable managing complicated chronic conditions of children who were being seen in establishments that have been locked down for COVID-19 patients	Always	33	12.5	**0.390** ^*∗*^
	Never	80	30.3	
	Sometimes	151	57.2	

Tried to contact the primary treating physician of patients under your care	No	79	29.9	**0.511** ^*∗*^
	Sometimes	76	28.8	
	Yes	109	41.3	

When do you decide to contact the primary treating physician?	No answers	6	2.3	**0.759** ^*∗*^
	I Did not try to contact the primary treating physician	66	25.0	
	It was easy as you have his/her contacts	114	43.2	
	It was impossible as he/she did not respond to your call	12	4.5	
	It was not easy as you did not have his/her contacts	66	25.0	

Did children with chronic diseases under your care have difficulties getting their prescription medications?	Always	38	14.4	**0.125** ^*∗*^
	Never	42	15.9	
	Sometimes	184	69.7	

During the lockdown period, were caregivers reluctant to seek medical care for their children?	Always	57	21.6	**0.128** ^*∗*^
	Never	13	4.9	
	Sometimes	194	73.5	

Fatalities you had to report among children under your care due to delay or lack of appropriate medical care in the period of the lockdown?	0	159	60.2	**0.001** ^£^
	1–5	82	31.1	
	>5	23	8.7	

Do you think pediatric patients' caregivers avoided going to the emergency department during the lockdown?	Most of the time	135	51.1	**0.169** ^*∗*^
	Never	23	8.7	
	Sometimes	106	40.2	

Opinion regarding the official choice of COVID-19 quarantine hospitals	Strongly disagree	71	26.9	**0.987** ^*∗*^
	Disagree	34	12.9	
	Neutral	77	29.2	
	Agree	36	13.6	
	Strongly agree	46	17.4	

In the future, if similar conditions face the health care system in Jordan, would you encourage:	I Support herd immunity no need to lock down health care facilities for COVID-19 patients	41	15.5	**0.055** ^*∗*^
	Implementing the same current protocol, and assign the same hospitals for COVID-19 patient quarantine	56	21.2	
	Try to pick other establishments, outside large cities with a less negative impact on patients	167	63.3	

^*∗*^No significant difference based on specialty, Supplementary Table 1. ^§^Significant difference according to level of practice, with week magnitude; Supplementary Table 2. ^£^Significant difference according to level of practice, with standardized residual absolute value > 3; Supplementary Tables 4–6.

## Data Availability

Data will be available upon request via e-mailing the corresponding author.

## Supplementary Materials

Supplementary Materials Supplementary Table 1: Questions that did not have significant differences based on specialty. Supplementary Table 2: Statistically significant differences in responses to the questionnaire with negligible relationship (Cramer's *V* < 0.19) based on practice level. Supplementary Table 3: Statistically significant differences in responses to the questionnaire, place of practice with specialty, moderate magnitude (Cramer's *V* > 0.3), and frequencies higher than expected (Standardized Residual > 3). Supplementary Table 4: Statistically significant differences in responses to the questionnaire, number of patients with specialty, moderate magnitude (Cramer's *V* > 0.3), and frequencies higher than expected (Standardized Residual > 3). Supplementary Table 5: Statistically significant differences in responses to the questionnaire, method of service provided with specialty, moderate magnitude (Cramer's *V* > 0.3), and frequencies higher than expected (Standardized Residual > 3). Supplementary Table 6: Statistically significant differences in responses to the questionnaire, fatalities reported according to specialty, weak magnitude (Cramer's *V* > 0.2–0.29), and frequencies higher than expected (Standardized Residual > 3).
