# Supplementary material for: Evaluation of the Effect Coronavirus Lockdown had on Chronic Disease Management Care in Pediatrics: A Survey of Jordanian Pediatricians
Source: Int J Clin Pract. 2022 Oct 6;2022:8710176. doi: 10.1155/2022/8710176 (PMC9560848; doi:10.1155/2022/8710176)
Supplement: Supplementary Materials — Supplementary Table 1: Questions that did not have significant differences based on specialty. Supplementary Table 2: Statistically significant differences in responses to the questionnaire with negligible relationship (Cramer's V < 0.19) based on practice level. Supplementary Table 3: Statistically significant differences in responses to the questionnaire, place of practice with specialty, moderate magnitude (Cramer's V > 0.3), and frequencies higher than expected (Standardized Residual > 3). Supplementary Table 4: Statistically significant differences in responses to the questionnaire, number of patients with specialty, moderate magnitude (Cramer's V > 0.3), and frequencies higher than expected (Standardized Residual > 3). Supplementary Table 5: Statistically significant differences in responses to the questionnaire, method of service provided with specialty, moderate magnitude (Cramer's V > 0.3), and frequencies higher than expected (Standardized Residual > 3). Supplementary Table 6: Statistically significant differences in responses to the questionnaire, fatalities reported according to specialty, weak magnitude (Cramer's V > 0.2–0.29), and frequencies higher than expected (Standardized Residual > 3). [file 8710176.f1.docx]

**Supplementary Table 1:** Questions that did not have significant differences based on specialty.

| Questions | Statistic Inferences |
| --- | --- |
| Was your facility locked down, for only treating COVID 19 patient role? | χ^2^(2) =5.710, p =0.058 |
| Do you have COVID-19 positive patient cases? | χ^2^(2) = 3.456, p =0.178. |
| In your current establishment, how has the number of patients seen in different departments changed in the period of the lockdown? | χ^2^ (8) = 10.367, p=0.240 |
| Have you seen children who have been already diagnosed with chronic illnesses? | χ^2^ (2) = 3.185, p=0.203. |
| Did you feel comfortable managing complicated chronic conditions of children who were being seen in establishments that have been locked down for COVID -19 patients (Prince Hamzah Hospital, Qween Alia military hospital, and King Abdallah Hospital)? | χ^2^ (4) = 4.119, p=0.390 |
| Have you ever tried to contact the primary treating physician of patients under your care? | χ^2^ (4) =3.289, p=0.511 |
| When do you decide to contact the primary treating physician? | χ^2^ (8) = 4.984, p=0.759 |
| Did children with chronic diseases under your care have difficulties getting their prescription medications? | χ^2^ (4) = 7.222, p=0.125 |
| During the lockdown period, Caregivers always reluctant to seek medical care for their children | χ2 (2) = 4.11, p=0.128 |
| Do you think pediatric patients’ caregivers avoided going to the Emergency Department during the lockdown? | χ^2^ (4) = 6.428, p=0.169 |
| Please state your opinion regarding choosing (Prince Hamzah Hospital, Qween Alia military hospital, and King Abdallah Hospital) as COVID-19 quarantine Hospitals. | χ^2^ (8) = 1.798, p=0.987 |
| In the future, if similar conditions face the health care system in Jordan, would you encourage | χ^2^ (4) = 9.277, p=0.055 |

**Supplementary Table 2:** Statistically significant differences in responses to the questionnaire with negligible relationship (Cramer’s V <0.19) based on specialty level.

| Questionnaire | Category | Consultant | Resident | Specialist | Total | Statistic Chi-square | Cramer's V | P-value |
| --- | --- | --- | --- | --- | --- | --- | --- | --- |
| Gender | Female | 41 | 25 | 48 | 114 | 18.06 | 0.185 | 0.001 |
|  |  | (43.2%) | (28.4%) | (59.3%) | 43.2% |  |  |  |
|  | Male | 53 | 63 | 32 | 148 |  |  |  |
|  |  | (55.8%) | (71.6%) | (39.5%) | 56.1% |  |  |  |
|  | Prefer not to answer | 1 (1.1%) | 0 (0.0%) | 1 (1.2%) | 2 (0.8%) |  |  |  |
|  |  |  |  |  |  |  |  |  |
| Did you practice pediatrics during the lockdown period? | No | 25  26.3% | 10  11.4% | 12  14.8% | 47 17.8% | 7.69 | 0.171 | 0.021 |
|  |  |  |  |  |  |  |  |  |
|  | Yes | 70  73.7% | 78  88.6% | 69  85.2% | 217 82.2% |  |  |  |
|  |  |  |  |  |  |  |  |  |
| How would you describe the health condition of most of the chronically ill children that you might have seen? | Controlled on given management | 32  33.7% | 15 17.0% | 18 22.2% | 65 24.6% | 15.62 | 0.172. | 0.016 |
|  |  |  |  |  |  |  |  |  |
|  | Not applicable | 4  4.2% | 11 12.5% | 13 16.0% | 28 10.6% |  |  |  |
|  |  |  |  |  |  |  |  |  |
|  | Stable, yet need closer to follow up | 42  44.2% | 47 53.4% | 43 53.1% | 132 50.0% |  |  |  |
|  |  |  |  |  |  |  |  |  |
|  | Uncontrolled with emerging complications | 17  17.9% | 15 17.0% | 7  8.6% | 39 14.8% |  |  |  |
|  |  |  |  |  |  |  |  |  |

**Supplementary Table 3:** Statistically significant differences in responses to the questionnaire, Place of practice with specialty, moderate magnitude (Cramer’s V >0.3), and frequencies higher than expected (Standardized Residual > 3).

|  |  | | Level of practice specialty | | | Total | Statistic Chi-square | Cramer's V | P-value |
| --- | --- | --- | --- | --- | --- | --- | --- | --- | --- |
|  |  |  | Consultant | Resident | Specialist |  |  |  |  |
|  |  | |  |  |  |  |  |  |  |
| Place of practice | Ministry of health | Count | 28 | 70 | 28 | 126 | 62.51 | 0.344 | <0.001 |
|  |  | % within level of practice | 29.5% | 79.5% | 34.6% | 47.7% |  |  |  |
|  |  | Standardized Residual | -2.6 | 4.3* | -1.7 |  |  |  |  |
|  | Private sector | Count | 37 | 10 | 28 | 75 |  |  |  |
|  |  | % within level of practice | 38.9% | 11.4% | 34.6% | 28.4% |  |  |  |
|  |  | Standardized Residual | 1.9 | -3.0 | 1.0 |  |  |  |  |
|  | Royal medical services | Count | 14 | 5 | 20 | 39 |  |  |  |
|  |  | % within level of practice | 14.7% | 5.7% | 24.7% | 14.8% |  |  |  |
|  |  | Standardized Residual | .0 | -2.2 | 2.3 |  |  |  |  |
|  | University Hospital | Count | 16 | 3 | 5 | 24 |  |  |  |
|  |  | % within level of practice | 16.8% | 3.4% | 6.2% | 9.1% |  |  |  |
|  |  | Standardized Residual | 2.5 | -1.8 | -.9 |  |  |  |  |
| * Standardized Residual with absolute value >3 | | | | | | | | | |

**Supplementary Table 4:** Statistically significant differences in responses to the questionnaire, Number of patients with specialty, moderate magnitude (Cramer’s V >0.3), and frequencies higher than expected (Standardized Residual > 3).

|  | | | Level of practice specialty | | | Total | Statistic Chi-square | Cramer's V | P-value |
| --- | --- | --- | --- | --- | --- | --- | --- | --- | --- |
|  |  |  | Consultant | Resident | Specialist |  |  |  |  |
| Number of patients per day you have seen during the lockdown | >30 | Count | 6 | 25 | 5 | 36 | 59.882 | 0.336 | <0.001 |
|  |  | % within level of practice | 6.3% | 28.4% | 6.2% | 13.6% |  |  |  |
|  |  | Standardized Residual | -1.9 | **3.8*** | -1.8 |  |  |  |  |
|  | 0 | Count | 20 | 1 | 11 | 32 |  |  |  |
|  |  | % within level of practice | 21.1% | 1.1% | 13.6% | 12.1% |  |  |  |
|  |  | Standardized Residual | 2.5 | -3.0 | .4 |  |  |  |  |
|  | 1-10 | Count | 52 | 21 | 36 | 109 |  |  |  |
|  |  | % within level of practice | 54.7% | 23.9% | 44.4% | 41.3% |  |  |  |
|  |  | Standardized Residual | 2.0 | -2.5 | .4 |  |  |  |  |
|  | 11-20 | Count | 13 | 26 | 20 | 59 |  |  |  |
|  |  | % within level of practice | 13.7% | 29.5% | 24.7% | 22.3% |  |  |  |
|  |  | Standardized Residual | -1.8 | 1.4 | .4 |  |  |  |  |
|  | 21-30 | Count | 4 | 15 | 9 | 28 |  |  |  |
|  |  | % within level of practice | 4.2% | 17.0% | 11.1% | 10.6% |  |  |  |
|  |  | Standardized Residual | -1.9 | 1.9 | .1 |  |  |  |  |
| * Standardized Residual with absolute value >3 | | | | | | | | | |

**Supplementary Table 5:** Statistically significant differences in responses to the questionnaire, Method of service provided with specialty, moderate magnitude (Cramer’s V >0.3), and frequencies higher than expected (Standardized Residual > 3).

|  | | | Level of practice | | | Total | Statistic Chi-square | Cramer's V | P-value |
| --- | --- | --- | --- | --- | --- | --- | --- | --- | --- |
|  |  |  | Consultant | Resident | Specialist |  |  |  |  |
| Most of the time, during the lockdown period, how did you provide your services to your patients? | Both ways | Count | 45 | 44 | 57 | 146 | 58.02 | 0.331 | <0.001 |
|  |  | % within level of practice | 47.4% | 50.0% | 70.4% | 55.3% |  |  |  |
|  |  | Standardized Residual | -1.0 | -.7 | 1.8 |  |  |  |  |
|  | In-person; face to face | Count | 17 | 43 | 11 | 71 |  |  |  |
|  |  | % within level of practice | 17.9% | 48.9% | 13.6% | 26.9% |  |  |  |
|  |  | Standardized Residual | -1.7 | 4.0 | -2.3 |  |  |  |  |
|  | Remotely; by phone, Facebook...... | Count | 33 | 1 | 13 | 47 |  |  |  |
|  |  | % within level of practice | 34.7% | 1.1% | 16.0% | 17.8% |  |  |  |
|  |  | Standardized Residual | **3.9** | -3.7 | -.4 |  |  |  |  |
| * Standardized Residual with absolute value >3 | | | | | | | | | |

**Supplementary Table 6:** Statistically significant differences in responses to the questionnaire, Fatalities reported with specialty, weak magnitude (Cramer’s V >0.2 – 0.29), and frequencies higher than expected (Standardized Residual > 3).

|  | | | Level of Practice | | | Total | Statistic Chi-square | Cramer's V | P-value |
| --- | --- | --- | --- | --- | --- | --- | --- | --- | --- |
|  |  |  |  | | |  |  |  |  |
|  |  |  | Consultant | Resident | Specialist |  |  |  |  |
| How many fatalities you had to report among children under your care due to delay or lack of appropriate medical care in the period of the lockdown? | >5 | Count | 3 | 17 | 3 | 23 | 29.38 | 0.236 | <0.001 |
|  |  | % within level of practice | 3.2% | 19.3% | 3.7% | 8.7% |  |  |  |
|  |  | Standardized Residual | -1.8 | 3.4* | -1.5 |  |  |  |  |
|  | 0 | Count | 72 | 39 | 48 | 159 |  |  |  |
|  |  | % within level of practice | 75.8% | 44.3% | 59.3% | 60.2% |  |  |  |
|  |  | Standardized Residual | 2.0 | -1.9 | -.1 |  |  |  |  |
|  | 1-5 | Count | 20 | 32 | 30 | 82 |  |  |  |
|  |  | % within level of practice | 21.1% | 36.4% | 37.0% | 31.1% |  |  |  |
|  |  | Standardized Residual | -1.8 | .9 | 1.0 |  |  |  |  |
| * Standardized Residual with absolute value >3 | | | | | | | | | |
